# Peptide Conjugated Boron Neutron Capture Therapy for Enhanced Tumor Targeting

**DOI:** 10.7150/ntno.95251

**Published:** 2024-05-28

**Authors:** Vaskuri G.S. Sainaga Jyothi, Nagavendra Kommineni

**Affiliations:** 1 Department of Pharmaceutical Sciences, University of Tennessee Health Science Center (UTHSC), Memphis, TN 38163, USA.; 2 Center for Biomedical Research, Population Council, New York, NY 10065, USA.

**Keywords:** BNCT, Peptide conjugation, Tumor targeting, targeting peptides and BNCT designing

## Abstract

A cutting-edge non-invasive cancer treatment method called boron neutron capture therapy (BNCT) allows for the removal of cancerous tumor cells with the least possible damage to healthy tissue. It involves the exposure of cancer cells with low-energy thermal neutrons, boron-10 (^10^B) cellular uptake causes cancer cell death by producing alpha particles and recoiling lithium-7 (^7^ Li) nuclei. Despite positive outcomes from clinical trials conducted all around the world, these substances have relatively limited tumor selectivity or low boron content per molecule. The development of new boron delivery agents with more selectivity and enhanced boron loading would advance this technique and promote its use in clinics as a primary cancer treatment. As peptide-binding cell surface receptors are typically overexpressed on cancer cells, they can be seen as interesting targets for targeted tumor therapy. The attachment of meta-carboranes to peptide conjugates that target tumor cells specifically by their overexpressed receptors may be a method to get around these problems. A state-of-the-art overview of current developments in the application of BNCT for cancer targeted therapy via peptide conjugation is the goal of this review.

## Introduction

Peptide conjugated boron neutron capture therapy (BNCT) is a type of cancer treatment that combines two elements: boron and neutron radiation. Boron is a non-toxic element that can be selectively taken up by cancer cells when delivered in the form of a boron-containing compound 1. Neutron radiation, on the other hand, can be used to trigger a nuclear reaction in boron atoms, releasing high-energy particles that can kill cancer cells 2. Peptide conjugated BNCT takes advantage of the fact that certain peptides can bind specifically to proteins that are overexpressed on the surface of cancer cells, such as receptors or transporters 3. By attaching boron-containing compounds to these peptides, it is possible to selectively deliver boron to cancer cells while sparing healthy tissue 4. The peptide conjugated BNCT process involves several steps. First, a boron-containing compound is conjugated to a targeting peptide. The resulting peptide-boron conjugate is then administered to the patient, where it circulates in the blood stream and selectively binds to cancer cells. Once the conjugate has accumulated in the tumor, the patient is exposed to a beam of neutrons, which causes boron atoms in the conjugate to undergo a nuclear reaction and release high-energy particles that kill nearby cancer cells 5-8.

Peptide conjugated BNCT has several potential advantages over other cancer treatments 9. It is highly specific, meaning that it can target cancer cells while sparing healthy tissue. It is also a non-invasive procedure, which means that it does not require surgery 10. Additionally, because boron compounds are not toxic at low concentrations, they can be administered at relatively high doses without causing significant side effects 11. However, there are also some challenges associated with peptide conjugated BNCT 12. Additionally, the neutron beam used to trigger the nuclear reaction must be carefully controlled to avoid damaging healthy tissue 13. Overall, peptide conjugated BNCT shows promise as a targeted cancer therapy, but further research is needed to fully understand its potential and optimize its effectiveness 14.

## Principles and BNCT's basic requirements

**Figure 1:**
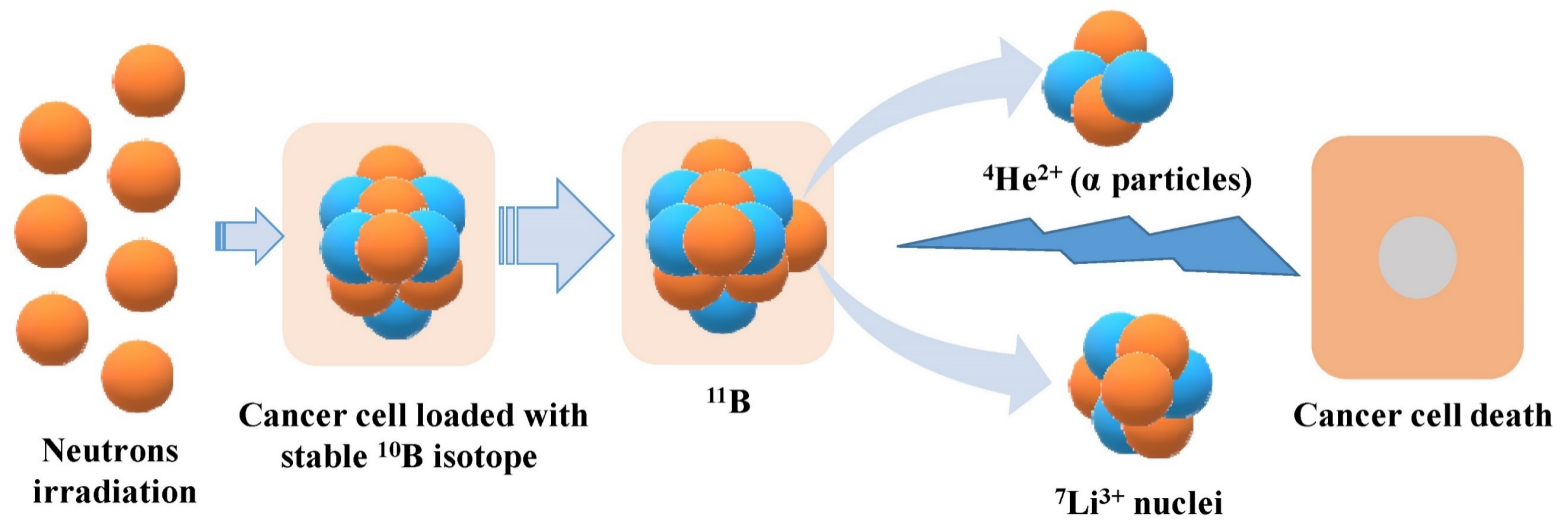
Neutron irradiated cancer cells containing boron compounds leads to cell death.

BNCT is a radiotherapy that involves two steps to achieve a selective radiation effect on tumor cells. Firstly, the tumor is targeted with non-radioactive boron (^10^B), followed by exposure to low-energy neutrons 15. The stable ^10^B isotope used has a large cross-sectional area for the capture of thermal neutrons, which then yield intensively ionizing particles ^4^He^2+^ (α particles) and recoiling ^7^Li^3+^ nuclei (**Figure 1**) 16. These nuclear fragments are highly cytotoxic and move very short distances within tissues where α particles travel for 9 µm and Li particles for 5 µm, destroying cells that have taken up the ^10^B containing compounds 17.

The low-energy neutron beam used in BNCT does not cause significant damage to tissues. However, the boron agent selected should have no toxicity. BNCT can be performed at an optimal time when the tumor/blood and tumor/surrounding tissue concentrations reach a favourable value, providing better control in selectively destroying tumor cells while minimizing damage to normal cells, unlike conventional chemotherapy or radiotherapy 18. Moreover, the neutron beam can be focused narrowly on brain tumors, preventing other organs that may have accumulated significant amounts of boron, like the liver and kidney, from being affected by the therapy 19.

The basic principles of BNCT rely on the ability of boron-containing compounds to accumulate selectively in cancer cells. This can be achieved through the use of boron-carrying drugs or by combining boron with specific targeting molecules such as peptides or antibodies 20,21. Once the boron has accumulated in cancer cells, the patient is exposed to a beam of thermal neutrons. These neutrons are absorbed by the boron atoms in the cancer cells, triggering a nuclear reaction that produces α particles and lithium ions. These high-energy particles then selectively destroy the cancer cells while sparing normal tissues 22. The success of BNCT depends on delivering a sufficient dose of radiation to the tumor while minimizing damage to healthy tissues 23. This requires careful selection of the boron compound, the neutron source, and the treatment regimen.

The basic requirements for BNCT include a boron containing compound, a neutron source and a treatment plan. A boron-containing compound that can selectively accumulate in cancer cells is needed for BNCT 24. The boron compound must have a high enough concentration of boron to effectively kill cancer cells, but not so high that it becomes toxic 25. A source of thermal neutrons is required for BNCT. This can be a nuclear reactor or a proton accelerator-based neutron source. Because BNCT uses high-energy radiation, careful shielding of both the patient and medical staff is necessary to minimize exposure to radiation 26. A thorough treatment planning process is required to optimize the BNCT dose delivery 27. This may involve imaging studies, such as MRI or CT scans, to accurately target the tumor and avoid nearby normal tissues 28-30. BNCT is a complex treatment that requires a team of experts, including radiation oncologists, medical physicists, and radiation safety specialists, to ensure safe and effective treatment delivery 31. Overall, BNCT is a promising treatment modality for certain types of cancer, but it requires careful planning and expertise to ensure optimal outcomes.

## Types of boron containing agents

**Figure 2:**
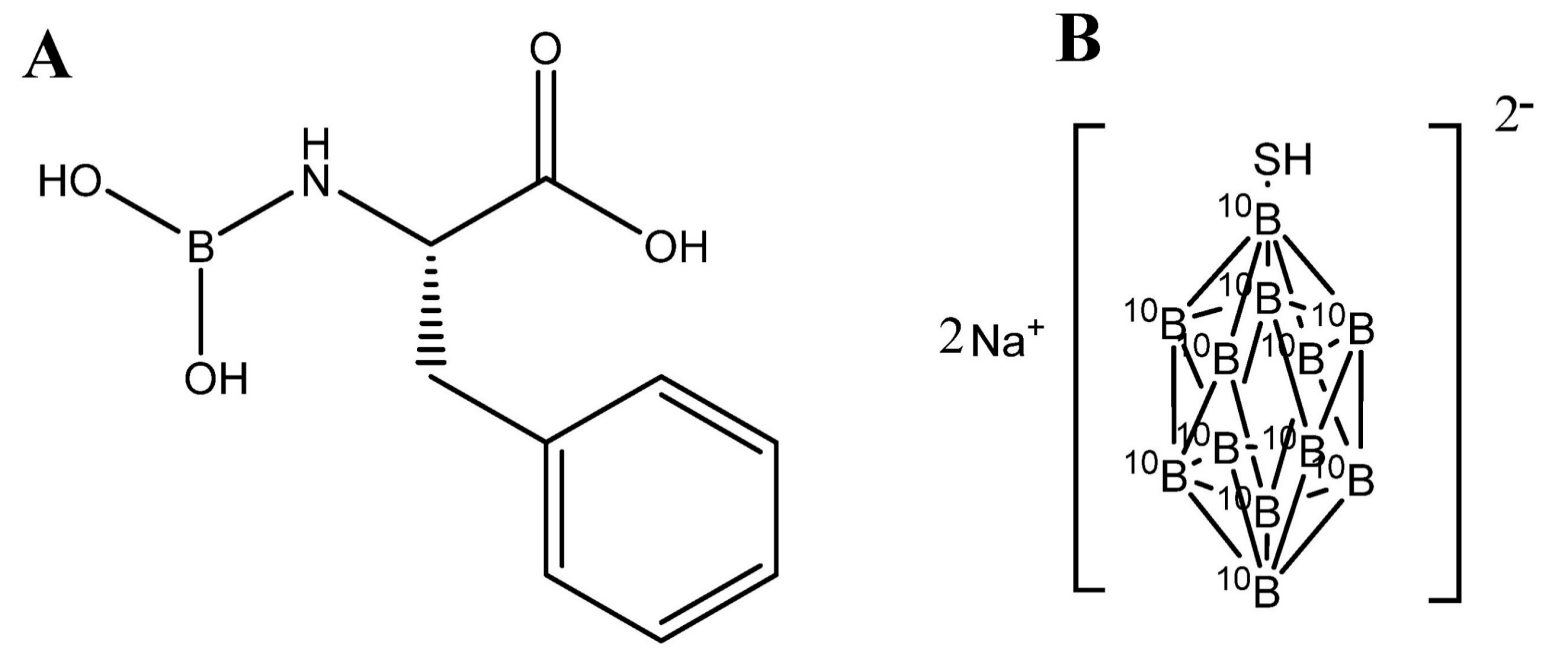
Chemical structures of (A) boronophenylalanine (BPA) and (B) sodium borocaptate (BSH)

Several boron-containing compounds have been developed for use in BNCT, including Boronophenylalanine (BPA) 32-34, sodium borocaptate (BSH) 35-37, boronated porphyrins 1,8,38. Boronophenylalanine (BPA) is a widely used compound, which comprises of an amino acid (**Figure 2A**). It has studied for different types of cancer including malignant brain tumors 39, malignant melanoma 32, malignant mesothelioma 40 and recurrent head and neck cancer 41. BPA localization in tumors is mediated by transporters. The altered metabolism in tumor cells favour the uptake of amino acid via upregulated amino acid transporters. BPA, being an amino acid analogue, internalized selectively in the tumors via the L transporter, specifically LAT1 transporter 42. It has been used in clinical trials for the treatment of brain tumors and melanoma, with promising results. On March 25, 2020, the Japanese Ministry of Health, Labour, and Welfare approved BPA as a boron delivery agent for BNCT of advanced or recurrent head and neck malignancies 43.

Sodium borocaptate (BSH) is being used for more than 50 years and used for the treatment of glioblastoma multiforme. It is not permeable to cross the blood brain barrier (BBB) and hence its concentration in the brain normal tissue is low 44. However, due to the breakdown of BBB, it permeable to accumulate in the malignant brain tumors 45. BSH is the compound having high boron content with the molecular formula (B_12_H_11_SH) Na_2_) (**Figure 2B**). However, it suffers from poor cellular uptake which halted its success as a potential candidate 37. Studies were reported to extend its viability, cell permeable compounds were tagged enhancing its cellular uptake and tissue retention. Futamura *et al.,* synthesized amino acid tagged BSH molecule. The amino acid used was 1-amino-3-fluorocyclobutane-1-carboxylic acid, which is actively taken up by cells with L-type amino acid transporters. The results showed high cellular uptake, biodistribution and high survival rate in F98 rat glioma cells as compared to BPA and BSH 46.

Boronated porphyrins are a class of compounds that contain boron atoms attached to a porphyrin backbone 47. Carboranyl porphyrins are a specific type of boronated porphyrin that contain multiple boron atoms attached to a carbon atom in the porphyrin ring 48. Because of their high boron content and ability to bind to cancer cells, carboranyl-substituted porphyrins, stable conjugates of macrocyclic porphyrins with intricate polyhedra containing boron, are regarded as promising BNCT candidates. Shi Y *et al.,* coated boronated porphyrins with a biocompatible poly(lactide- co-glycolide)-monomethoxy-poly(polyethylene-glycol) (PLGA-mPEG) micelle which aided in accumulation in tumor selectively thereby reducing toxicity as compared with other boronated porphyrins 49.

Overall, while some boron-containing compounds have shown promise in preclinical and clinical studies, further research is needed to evaluate their efficacy and safety in humans. The development of new boron-containing compounds and improved delivery methods may lead to more effective and targeted BNCT treatments in the future 50.

## Clinical trials of BNCT

Numerous clinical trials were performed on BNCT compounds to treat various cancers and are reported in **Table 1**. The effectiveness of surgery and traditional chemo-radiotherapy is limited in patients with recurrent squamous cell carcinoma (SCC) and locally advanced non-squamous cell carcinoma without malignant melanoma (non-SCC) of the head and neck. Clinical trials of BNCT for head and neck cancers are currently being conducted in various institutions to determine its efficacy 51,52.

Between October 2003 and September 2007, BNCT on 10 patients with recurrent SCC, 7 patients with recurrent non-SCC, and 3 patients with newly diagnosed non-SCC were performed. Of these patients, 11 showed complete remission and 7 showed partial remission at the irradiated site. The overall effective rate, calculated as the sum of complete and partial remission cases divided by the total number of cases, was 90%. Furthermore, no severe acute or chronic normal-tissue reactions were observed in any of the patients, indicating that BNCT is a safe and effective treatment option for patients with recurrent SCC and locally advanced non-SCC 53.

Another clinical trial of recurrent head-and-neck cancer using BNCT at Tsing Hua open-pool reactor (THOR) was conducted. The THOR beam provides a high-quality epithermal neutron beam for BNCT in Taiwan. The treatment plans were performed using the in-house treatment planning system THOR plan, and the dose calculations were carried out by Monte Carlo N-Particle Transport code, MCNP. The boron drug used was boronophenylalanine-fructose (BPA-F) with enriched boron. Intravenous L-BPA-F 400 mg/kg was administered in 2 phases. Two different irradiation conditions on patients 16 and 17 were performed; direct irradiation and irradiation with a patient collimator. The use of the patient collimator resulted in an increased thermal neutron flux near the skin surface and inside the collimator, while decreasing the neutron flux outside the collimator. This approach was found to be particularly beneficial for superficial tumor treatment. The use of a patient collimator resulted in a reduced irradiation time for patient 17 and a slightly increased irradiation time for patient 16. The maximum dose rate of mucosa was reduced in patient 17 and slightly reduced in patient 16 when a patient collimator was used. The maximum dose rate of brain and skin increased slightly in both patients when a patient collimator was used. Superficial tumor treatment gains benefits from the use of patient collimator, while direct irradiation is a better choice for deep-seated tumors 54.

A study to assess the safety and efficacy of fractionated BNCT for recurrent head and neck (H&N) cancer after photon radiation therapy was performed. In this phase 1/2 trial, two-fraction BNCT was administered with intravenous L-boronophenylalanine (L-BPA, 400 mg/kg) at a 28-day interval. Prior to each fraction, fluorine-18-labeled-BPA-positron emission tomography was performed to determine the tumor/normal tissue ratio of each individual tumor. A prescription dose (D80) of 20 Gy-Eq per fraction was chosen to cover 80% of the gross tumor volume using a dose volume histogram while minimizing the volume of oral mucosa receiving >10 Gy-Eq. Response Evaluation Criteria in Solid Tumors v1.1 and the Common Terminology Criteria for Adverse Events v3.0 were used to assess tumor response and adverse effects, respectively 55.

Seventeen patients with a previous cumulative radiation dose of 63-165 Gy were enrolled. All but two participants received two fractions of BNCT. The median tumor/normal tissue ratio was 3.4 for the first fraction and 2.5 for the second, while the median D80 for the first and second fraction was 19.8 and 14.6 Gy-Eq, respectively. After a median follow-up period of 19.7 months (range, 5.2-52 months), 6 participants exhibited a complete response, and 6 exhibited a partial response. Regarding acute toxicity, 5 participants experienced grade 3 mucositis, and 1 participant experienced grade 4 laryngeal edema and carotid hemorrhage. Regarding late toxicity, 2 participants developed grade 3 cranial neuropathy. Four out of six participants (67%) receiving total D80 > 40 Gy-Eq achieved a complete response. Two-year overall survival was 47%, while two-year locoregional control was 28%.

The results indicated that two-fraction BNCT with adaptive dose prescription was a safe and effective treatment for locally recurrent H&N cancer. Modifications to the protocol may lead to better results in the future 55.

A clinical study was performed which explored the differences in dose distribution in the gross tumor volume (GTV) between two treatment modalities for head and neck cancer: BNCT alone and combined BNCT plus intensity modulation radiation therapy (IMRT). The study involved nine patients with recurrent head and neck cancer who were treated with BNCT at the Tsing-Hua Open pool reactor using the THOR plan treatment planning system. The aim was to investigate whether intensity modulated radiation therapy (IMRT) could be used to address the dose heterogeneity observed in GTV with single-fraction BNCT. The study found that the BNCT+IMRT plan provided better conformity index and homogeneity in the GTV than the BNCT-alone plan. The inhomogeneous dose distribution in the BNCT plan resulted in cold spots, which may contribute to cancer recurrence. The study suggests that combining single-fraction BNCT with compensated multi-fraction IMRT can improve treatment homogeneity and conformity, especially for tumor volumes >100 cm^3^, and potentially increase local tumor control 56.

Another study was performed to assess the safety and effectiveness of cyclotron-based BNCT with borofalan (^10^B) for patients with recurrent or locally advanced head and neck cancer. BNCT was performed without the need for reactors due to the development of a cyclotron-based epithermal neutron source (C-BENS), which is optimized for deeper-seated tumors. In this open-label, phase II JHN002 trial, patients with recurrent squamous cell carcinoma (R-SCC) or with recurrent/locally advanced non-squamous cell carcinoma (R/LA-nSCC) of the head and neck were administered 400 mg/kg borofalan (^10^B) intravenously, followed by neutron irradiation using C-BENS. The primary endpoint was the objective response rate (ORR), and the post-trial observational JHN002 look up study was planned for evaluating locoregional progression-free survival (LRPFS).

A total of eight R-SCC and 13 R/LA-nSCC patients were enrolled, all of whom had previously undergone radiation therapy, with a median dose of 65.5 Gy (range, 59.4-76.0 Gy) for R-SCC patients. The ORR for all patients was 71%, with complete response/partial response rates of 50%/25% in R-SCC and 8%/62% in R/LA-nSCC. The 2-year overall survival rates for R-SCC and R/LA-nSCC were 58% and 100%, respectively. The median LRPFS was 11.5 months for R-SCC patients. The most observed adverse events were alopecia (95%), hyperamylasemia (86%), and nausea (81%). The data suggest that BNCT using C-BENS with borofalan (10B) is a promising treatment option for patients with R-SCC or R/LA-nSCC of the head and neck. This study provides evidence of the efficacy and safety of BNCT as a treatment option for these patients 57.

Although wide surgical excision is the most recommended treatment for genital melanoma and extramammary Paget's disease (EMPD), it can be highly invasive and lead to functional and sexual problems. Alternative treatments have been used when excision was not feasible. This study reports on four patients with genital malignancies who were treated with BNCT.

The patients included one with vulvar melanoma (VM) and three with genital EMPD. They underwent BNCT at the Kyoto University Research Reactor between 2005 and 2014 using para-boronophenylalanine as the boron delivery agent. They were irradiated with an epithermal neutron beam between the curative tumor dose and the tolerable skin/mucosal doses.

All patients showed similar tumor and normal tissue responses following BNCT and achieved complete responses within 6 months. The most severe normal tissue response was moderate skin erosion during the first 2 months, which gradually diminished. Dysuria or contact pain persisted for 2 months and resolved completely by 4 months.

Based on these results, BNCT is a promising treatment for primary VM and EMPD of the genital region. BNCT resulted in complete local tumor control in these four patients. The clinical experience suggests that BNCT can be a viable alternative treatment for genital malignancies 58.

A study was conducted to assess the dynamic changes in ^18^F-borono-L-phenylalanine (^18^F-BPA) uptake during BNCT therapy patient selection for unresectable, advanced, or recurrent squamous cell carcinoma of the head and neck (SCC) and malignant melanoma (MM). Changes in the maximum standardized uptake value (SUVmax), tumor-to-normal tissue ratio (TNR), and tumor-to-blood pool ratio (TBR) for ^18^F-BPA in 20 patients with SCC and 8 patients with MM was evaluated.

The results showed a significant decrease in SUVmax for SCC tumors from 30 to 120 minutes, with a non-statistically significant decrease from 30 to 60 minutes and from 60 to 120 minutes. However, patients with MM did not demonstrate any significant changes in ^18^F-BPA uptake on delayed imaging. Furthermore, no significant changes in ^18^F-BPA TNR and TBR in patients with SCC and MM was observed. Based on the findings, it was concluded that dynamic changes in SUVmax for ^18^F-BPA uptake follow a washout pattern in SCC and a persistent pattern in MM 59.

Accurately estimating tissue concentrations of ^10^B, particularly in neighboring normal organs, is crucial in BNCT for cancer to avoid adverse effects. However, the concentration of ^10^B in normal organs following loading with ^10^B has yet to be established in humans. To address this gap, a study was conducted where 4-borono-2-[^18^F]-fluoro-phenylalanine (^18^FBPA) and PET were used to estimate chronological changes in the ^10^B concentrations of normal organs in healthy volunteers.

Six healthy volunteers underwent whole-body ^18^FBPA PET scans, which were repeated seven times over the course of one hour, and the mean ^18^FBPA distributions of 13 organs were measured. Based on the ^18^FBPA PET data, the changes in the ^10^B concentrations of the organs were estimated assuming the injection of a therapeutic dose of ^10^BPA-fructose complex (^10^BPA-fr; 30 g, 500 mg/kg body weight).

The results showed that the maximum mean ^18^FBPA concentrations were reached at 2-6 minutes after injection in all organs except the brain and urinary bladder. The mean ^18^FBPA concentration in the normal brain plateaued at 24 minutes after injection. When assuming the injection of a therapeutic dose of ^10^BPA-fr, it was found that the estimated mean ^10^B concentration in the kidney increased to 126.1 ± 24.2 ppm at 3 minutes after injection and rapidly decreased to 30.9 ± 7.4 ppm at 53 minutes. The estimated mean ^10^B concentration in the bladder gradually increased and reached 383.6 ± 214.7 ppm at 51 minutes. The mean ^10^B concentration in the brain was estimated to be 7.6 ± 1.5 ppm at 57 minutes. In conclusion, the study suggested that ^18^FBPA PET has the potential to estimate the ^10^B concentration of normal organs before neutron irradiation of BNCT, provided that several assumptions are validated in future studies 60. These clinical studies showed the potency of BNCT in cancer therapy. However, the efficiency of the therapy can be improved by inculcating targeting strategy with the BNCT therapy for cancer treatment.

## Targeting peptide receptors in cancer therapy

Peptide conjugated BCNT is being evolved as a novel strategy for the targeted delivery of cancer therapeutics. Peptide-binding receptors play a crucial role in cancer therapy as they offer a specific and selective target for delivering anti-cancer agents 61-65. Peptide receptors are proteins on the surface of cells that bind to specific peptides, which can then be targeted with therapeutic agents 66-70. Conjugating peptide to the BNCT leads to a targeted therapy that use a peptide molecule to specifically deliver boron compounds to cancer cells. The peptide component is designed to bind to a specific peptide receptor on the surface of cancer cells, which allows for selective delivery of the drug payload to cancer cells while sparing normal cells 71-73. By conjugating boron-containing compounds to peptides that selectively bind to these receptors, peptide conjugated BNCT can deliver high doses of radiation to cancer cells while sparing normal tissues.

Several peptide receptors have been identified as potential targets for cancer therapy, including somatostatin receptors 74,75, gastrin-releasing peptide receptors 76-78, and integrin receptors 79,80. Somatostatin is a peptide hormone that regulates the release of other hormones in the body and regulates various physiological processes, including the secretion of growth hormone, insulin, and glucagon. Somatostatin receptors are overexpressed in a variety of tumors, including neuroendocrine tumors and small cell lung cancer. Peptides that bind to somatostatin receptors, such as octreotide and lanreotide, can be conjugated with boron compounds to aid in targeted delivery of BNCT and can selectively deliver high doses of radiation to cancer cells that express somatostatin receptors. Another example of a peptide receptor that can be targeted is the integrin receptor. Integrins are proteins that are involved in cell adhesion and signaling. They are overexpressed on the surface of many types of cancer cells and play a role in cancer progression and metastasis. By conjugating boron-containing compounds to peptides that bind to integrin receptors, BNCT can selectively deliver high doses of radiation to cancer cells that express these receptors 81. Peptides that bind to integrin receptors, such as RGD peptides, have been conjugated with BNCT to create peptide conjugated BNCT that specifically target tumor cells expressing integrin receptors.

In addition to somatostatin and integrin receptors, other peptide-binding receptors that have been targeted in cancer therapy include the folate receptor, the gastrin-releasing peptide receptor, and the epidermal growth factor receptor. Conjugation of BNCT with peptides that target these receptors grabbed the attention of scientific fraternity and research being conducted in this arena. Overall, targeting peptide-binding receptors in BNCT therapy using peptides is a promising approach to delivering boron compounds directly to cancer cells while minimizing damage to healthy tissues. BNCT compounds can be targeted to the cancerous cells by using various vectors or ligands including nucleosides, proteins, porphyrins and antibodies apart from peptides. However, targeting delivery with the peptides is more promising as the peptides are more specific to the receptors or antigens present on the surface of the cell where the other ligands or vectors lacks this target specificity leading to the off-target side effects. The peptides also aid in enhanced cellular uptake facilitated by the receptor-mediated endocytosis. The peptides are also smaller in size and simple with few amino acids as compared to other targeting ligands or vectors. Further research and development in this area may lead to more effective and personalized cancer therapies in the future 82.

## Applications of peptide conjugated BNCT

Peptide conjugated BNCT has several potential applications in cancer treatment (**Table 2**) 83. By conjugating boron-containing compounds to peptides that target specific proteins overexpressed on the surface of cancer cells, BNCT can selectively deliver high doses of radiation to cancer cells while sparing normal tissues. Thus, BNCT can be delivered to the cancer tissue by active targeting where the peptide enables specific deliver to the target site. The conjugated peptides also improve the cellular uptake of BNCT by enabling the receptor mediated endocytosis, thus, the peptide conjugates to the BNCT improved the delivery efficiency by overcoming the biological barriers. Based on the delivering vehicle for peptides conjugated BNCT, the size varies where the size < 100 nm is preferable for the tumor accumulation. Nakase *et al.,* studied a new approach to BNCT that Z33 peptide which shows binding affinity to the Fc receptor on cancer cells, which is overexpressed in many types of cancer. Z33 peptide is an Fc-binding peptide dodecaborate conjugate (Fc-BPC) was conjugated to boron-containing compounds, which were taken up by the cancer cells via macropinocytosis, a form of cellular uptake. The boron-containing compounds were then activated by neutron irradiation, leading to the destruction of the cancer cells. The study found that the Fc-BPC conjugate was highly effective in targeting cancer cells *in vitro* and *in vivo*, leading to increased uptake of boron-containing compounds and improved therapeutic efficacy in mouse models of cancer. The authors suggest that this approach has the potential to improve the specificity and efficacy of BNCT, particularly for cancers that overexpress the Fc receptor 84.

A study was reported to target tumor vasculature with IFLLWQR (IF7)-conjugated ^10^BPA or borocaptate sodium (^10^BSH). The researchers developed an Annexin A1-binding peptide that selectively binds to the tumor vasculature and conjugated it with a boron-containing compound for use in BNCT. In mouse models of cancer, the peptide conjugated BNCT approach was found to be highly effective in selectively targeting the tumor vasculature and delivering boron to the tumor cells. The treatment led to a significant reduction in tumor growth and increased survival rates compared to controls. The researchers suggest that this approach has the potential to improve the specificity and efficacy of BNCT, particularly for cancers that have a highly vascularized tumor microenvironment 85.

Another study presented a new approach to BNCT using a selective carborane-functionalized gastrin-releasing peptide receptor (GRPR) agonist as a boron delivery agent. The researchers developed bis-deoxygalactosyl-carborane, a carborane-functionalized GRPR agonist that selectively binds to GRPR 83, a receptor that is overexpressed in many types of cancer 101. *In vitro* and *in vivo* studies demonstrated that the carborane-functionalized GRPR agonist was highly selective and effective in targeting cancer cells that overexpress GRPR, leading to increased uptake of boron-containing compounds and improved therapeutic efficacy in mouse models of cancer 83.

Human Y1 receptor (hY1R) which is a type of GPCR 102 is targeted in another research where a selective neuropeptide Y (NPY) conjugated with bis-deoxygalactosyl-carborane, a boron delivery agent for BNCT was developed. The researchers aimed to optimize the boron loading of the conjugates while maintaining selectivity for the NPY receptor, which is overexpressed in many types of cancer. The researchers synthesized several NPY analogs conjugated with carborane, a boron-containing compound, and evaluated their boron loading, selectivity, and therapeutic efficacy. They found that optimizing the linker length and position of the carborane moiety resulted in NPY conjugates with maximized boron loading and selectivity for the NPY receptor. *In vitro* and *in vivo* studies demonstrated that the optimized NPY conjugates were highly selective and effective in targeting cancer cells that overexpress NPY receptor, leading to increased uptake of boron-containing compounds and improved therapeutic efficacy in mouse models of cancer 86.

Cell penetrating peptides (CPP) were also conjugated with the boron containing compounds to enhance uptake and penetration of boron compounds. Octa-arginine (R8) is a typical arginine-rich CPP was reported for targeted delivery of boron-containing compounds to intracellular targets. R8 is a peptide that is actively taken up via micropinocytosis and this CPP and RLA peptide (mitochondria-targeted peptide) was conjugated to dodecaborate as DB-RLA (BODIPY) and DB-r8 (BODIPY) respectively for targeted delivery. The researchers aimed to improve the therapeutic efficacy of BNCT by enhancing the intracellular delivery of boron-containing compounds. Using C6 glioma cells, the study examined the effects of CPP conjugation on the cellular uptake, cytosolic release, and intracellular localization of dodecaborate. Confocal laser microscopy demonstrated that DB-r8 displayed cytosolic/nuclear signals and endosome-like spots, while DB-RLA displayed mitochondrial accumulation. The cytosolic release of DB-r8 and the buildup of DB-RLA in the mitochondria were verified by antibody tests. When compared to unmodified BSH, quantitative analysis utilizing the enzyme-linked immunosorbent assay (ELISA) showed a four-fold increase in cellular uptake with RLA conjugation, but r8 conjugation inhibited cellular uptake. Following CPP-conjugated dodecaborate treatment, cellular viability was unaltered revealed by WST-1 assay after treatment with each of the CPP-conjugated dodecaborates (30 min, 37 ºC) 87.

In another study, angiopep-2 was used as CPP where angiopep-2 was conjugated with N-terminal boron, named ANG-B. Six distinct cancer cell lines were used to assess the novel compound, called ANG-B, *in vitro*. The conjugate showed improved cellular uptake and *in vivo* biodistribution, especially in HS683 glioma cell models. The findings emphasized the potential need for customized BNCT that is appropriate for particular tumor types by highlighting the variations in preferences among cell lines for various boron delivery agents. The results of the study supported the hypothesis that peptides with the capacity to penetrate cells are useful agents for the precise administration of boron-10 in BNCT, a treatment for cancer. Promising as a BNCT agent, ANG-B demonstrated effective uptake by cancerous cells and accumulation within tumors. ANG-B outperformed the commonly used BPA in controlling tumor growth in an intracranial glioma mouse model 103.

CPP is also conjugated with BSH whereby incorporating BSH into a cell-penetrating peptide (CPP) method allowed for effective transduction into cells, overcoming its restriction in intracellular localization. In comparison to traditional BSH administration under neutron irradiation, the synthesized 8BSH-11R, a CPP-fused boron molecule, showed superior intracellular localization and a high boron value, which resulted in notable cancer-killing effects. According to the study, BSH-fused CPPs, including 8BSH-11R, have better efficacy and may have clinical uses, making them promising for the next phase of BNCT studies. In particular, the study emphasizes the necessity for sophisticated boron compounds that go beyond the present agents like BSH in order to address the problems in BNCT. Using their distinct qualities, the combination of BPA and BSH is emphasized as being essential for the efficacy of glioblastoma treatment. The study suggests a novel, cost-effective, and safe method of delivering boron clusters into cells via CPPs. In particular, at low concentrations of BSH-peptide, the 8BSH-11R molecule exhibits significant tumor-killing activities, indicating its possible use as a central boron agent in BNCT down the road. By using functional peptides to target tumors, BNCT becomes a more viable and safe treatment option, establishing it as a significant cancer treatment of the future. The study comes to the conclusion that BNCT, a prominent cancer treatment, may advance considerably with the help of this novel boron compound with functional peptide integration 104.

Translocator protein (TSPO) ligand is highly expressed in most of the cancer including breast cancer. Hence a study was reported to target TSPO ligand using TSPO conjugating with the dodecaborate. The study aimed to improve the specificity and efficacy of BNCT by selectively targeting cancer cells that overexpress TSPO. The researchers synthesized a series of dodecaborate conjugates that selectively bind to TSPO and evaluated their cellular uptake and intracellular distribution *in vitro*. They found that the dodecaborate conjugates were highly effective in targeting cancer cells that overexpress TSPO, resulting in enhanced therapeutic efficacy *in vitro* 88.

Pteroyl-closo-dodecaborate-conjugated 4-(p-iodophenyl) butyric acid (PBC-IP), a cutting-edge boron agent designed for BNCT to overcome difficulties in the care of glioblastoma patients. PBC-IP has albumin-binding moieties, FRα-targeting, and a large 10B resource. PBC-IP exhibits glioma cell selectivity and surpass BPA at a much higher rate, especially in the U87MG xenograft model where it effectively inhibits tumor growth after neutron irradiation. In the F98 glioma orthotopic rat model, PBC-IP is administered via convection-enhanced delivery (CED). This results in a spectacular buildup of tumors and a 50% survival rate for 180 days following BNCT. When BPA and PBC-IP are combined, the survival rate rises to 70% and any remaining brain tumors are eradicated. More glioma cells accumulate in PBC-IP than in BPA, which makes it a viable substitute boron carrier for BNCT that may lower overall doses and side effects 105.

A self-assembling nanotube were developed with the peptide which also acted as delivery system. BSH was used a boron containing compound and A6K peptide was used to prove intracellular localization. A6K peptide nanotubes were synthesized and evaluated their ability to self-assemble and encapsulate BSH *in vitro*. The size of the nanotubes was determined by SEM found to be 522.5 ± 92.2 nm as long dimensions and 229.4 ± 43.9 nm as short dimensions. The study found that the A6K peptide nanotubes were highly effective in self-assembling and encapsulating BSH, resulting in enhanced solubility and stability of BSH. *In vitro* studies using cancer cells demonstrated that the A6K peptide nanotubes encapsulating BSH were highly effective in targeting and killing cancer cells when exposed to neutron irradiation. Brain tumor model in mouse demonstrated that high brain localization of A6K peptide conjugated BSH as compared to the BSH 9.

RGD peptide was explored as targeting ligand for BNCT where a study was aimed to develop RGD peptide-conjugated dodecaborate with the Ga-DOTA complex as a theranostic agent for BNCT and its companion diagnostics. The researchers synthesized RGD peptide-conjugated dodecaborate with the Ga-DOTA complex and evaluated its stability and ability to target cancer cells *in vitro*. The RGD-B12 conjugate was also evaluated *in vitro* for its binding affinity to integrin αvβ3 receptors using competitive binding assays, and *in vivo* using PET/CT imaging in a mouse tumor model. They found that the complex was highly stable and effectively targeted cancer cells that overexpressed integrin receptors. The results showed that the RGD-B12 conjugate exhibited high affinity and selectivity to integrin αvβ3 receptors both *in vitro* and *in vivo*. The PET/CT imaging study demonstrated significant tumor uptake of the RGD-B12 conjugate, indicating its potential as a theranostic agent for BNCT and its companion diagnostics 93.

Cyclic RGD was functionalized in a study reported by Chen J *et al.,* where a cyclic RGD-functionalized closo-dodecaborate albumin conjugates (cRGD-MID-BSA) as boron carriers for BNCT was synthesized. The research was aimed to enhance the therapeutic efficacy of BNCT by improving the targeting of boron to tumor cells using integrin-targeting cyclic RGD peptides.

**Figure 3:**
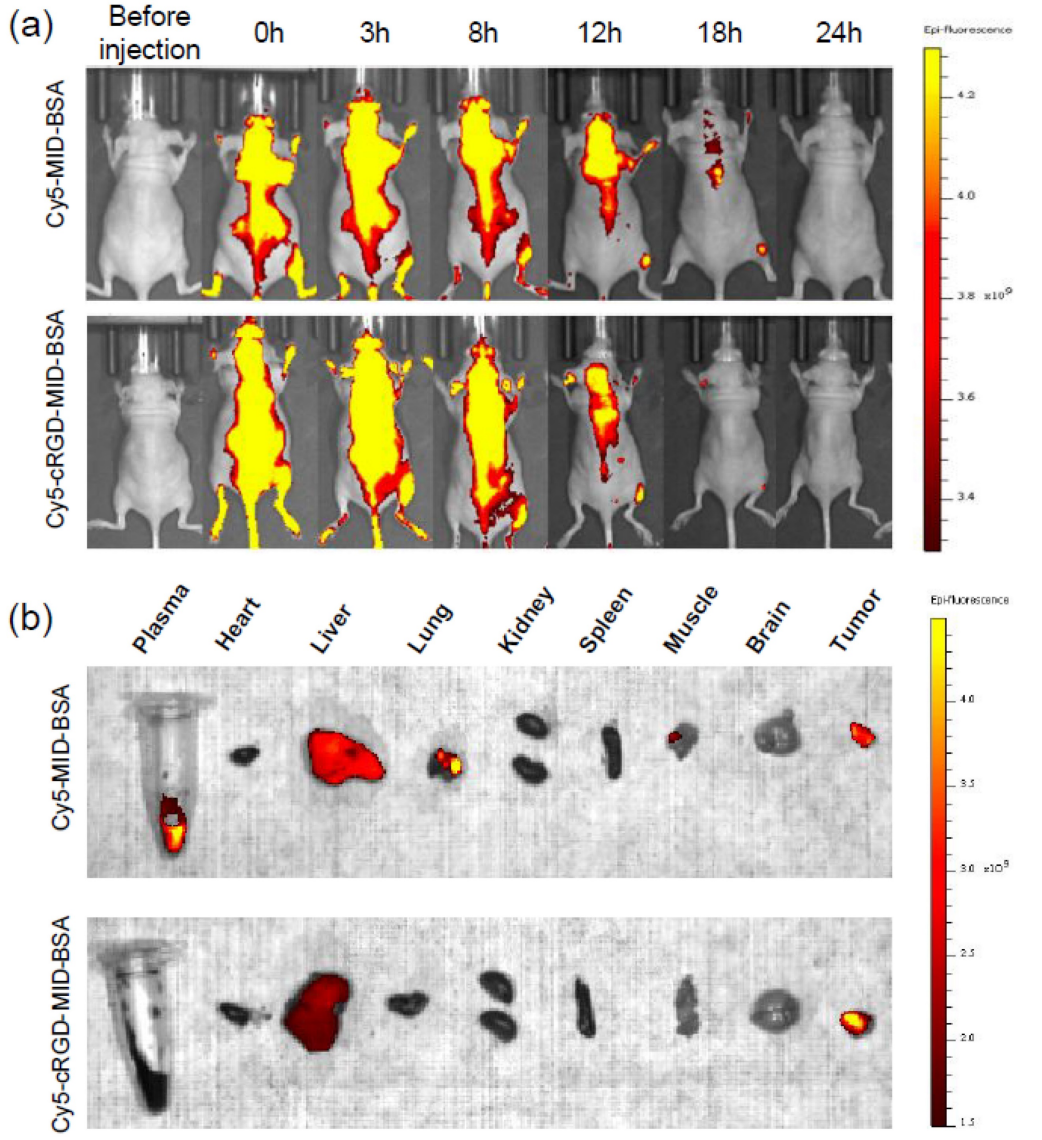
Fluorescence imaging of U87MG human glioblastoma xenograft tumors in female mice was conducted using Cy5-conjugated cRGD-MID-BSA and MID-BSA. (a) Live images were taken 24 hours post-injection, and (b) ex vivo images of various organs were captured 24 hours after injection. Reprinted (adapted) with permission from 91. Copyright {2024} American Chemical Society.

The *in vitro* stability and targeting ability of the conjugates was evaluated and found that cRGD-MID-BSA was effectively targeted αvβ3 integrin-expressing cells. The therapeutic efficacy of the conjugates was significantly enhanced compared to unmodified albumin conjugates and free boron compound. The *in vivo* biodistribution studies were performed in mice grafted with U87MG human glioblastoma cells. Cy5 tagged cRGD-MID-BSA and MID-BSA were iv injected and the fluorescent signals were observed in tumor and not in other organs with cRGD-MID-BSA. In contrast, along with tumor, liver, plasma and lungs showed signals with MID-BSA (**Figure 3**). The study suggested that cyclic RGD-functionalized closo-dodecaborate albumin conjugates have the potential to serve as an effective boron carrier for BNCT, providing improved targeting and therapeutic efficacy for cancer patients 91.

Polymeric nanoparticles were synthesized by modification with iRGD to improve the targeting and therapeutic efficacy of BNCT. PEG-PCCL polymer nanoparticles were synthesized with BNCT compounds, and the surface of the nanoparticles were modified with iRGD. The surface modified nanoparticles were stable at physiological pH and showed enhanced blood circulation and fivefold improvement in cellular uptake followed by improved tumor accumulation. The therapeutic efficacy of the nanoparticles was significantly enhanced compared to unmodified nanoparticles and free boron compound 90.

In another study, integrin αvβ3, a protein that is overexpressed in many types of cancer cells, was targeted with cyclic arginine-glycine-aspartate (cRGD) by using maleimide-functionalized closo-dodecaborate albumin conjugate (MID-AC) with albumin. Rat F98 glioma brain tumor model was used to evaluate the effectiveness of the new boron carrier conjugated with a peptide. The results of the study showed that the integrin αvβ3-targeted boron carrier had a higher tumor-to-blood ratio than the non-targeted boron carrier, indicating a higher accumulation of the boron carrier in the tumor. The researchers also found that the targeted boron carrier had a longer retention time in the tumor than the non-targeted carrier, which may lead to a higher radiation dose delivered to the tumor 94.

Miyabe, J *et al.,* studied the dipeptides of BPA and tyrosine targeting the oligopeptide transporter PEPT1. Oligopeptide transporters are expressed in various cancers. BPA was tagged with tyrosine as BPA-Tyr and Tyr-BPA. Both the BPA dipeptides were transported through transporters PEPT1 and PEPT2 respectively. AcPC-1 pancreatic cancer cells, expressed with PEPT-1, were treated with BPA-Tyr and Tyr-BPA and the results showed the delivery of boron was mediated with PEPT-1 mechanism. AsPC-1 xenograft tumors in mice showed accumulation of boron which could be due to the peptide tagged BPA 95.

Polyarginine conjugated boron containing compounds were studied in research. The study investigated the ability of polyarginine-conjugated sodium borocaptate (BSH-11R) to deliver boron to cancer cells. Polyarginine is a cell-penetrating peptide that can facilitate the uptake of molecules into cells. The study explored used various techniques, including flow cytometry and confocal microscopy, to study the uptake and subcellular localization of the polyarginine-conjugated sodium borocaptate in cancer cells. Overall, this study provides valuable insights into the potential of polyarginine-conjugated sodium borocaptate as a delivery agent for BNCT 37.

A novel approach to developing peptide agonists for the ghrelin receptor, a potential target for the treatment of obesity and metabolic disorders was reported. Boron-rich peptide agonist for the ghrelin receptor, which involves replacing a histidine residue with a meta-carborane compound was synthesized. The results of the study showed that the boron-rich peptide agonist was able to activate the ghrelin receptor, indicating its potential as a therapeutic agent. Additionally, the study demonstrated that the boron-rich peptide agonist was stable in serum and had a longer half-life than the original histidine-containing peptide agonist 97.

A study was performed on enzymes for targeting boron to the cancer cells which described the use of enzyme-instructed supramolecular assemblies (EISA) to increase intracellular boron accumulation. EISA involves the use of a small molecule prodrug that is activated by an enzyme, resulting in the formation of supramolecular assemblies that can selectively accumulate in cancer cells. EISA prodrug consists of a boron-containing compound conjugated to a hydrophobic tail and a peptide sequence that can be cleaved by an enzyme. The results of the study showed that the EISA prodrug was able to increase intracellular boron accumulation in cancer cells and enhance the effectiveness of BNCT *in vitro*. Thus the study concluded that the accumulation of boron mediated by EISA could be a promising strategy for the BNCT treatment 98.

**Figure 4:**
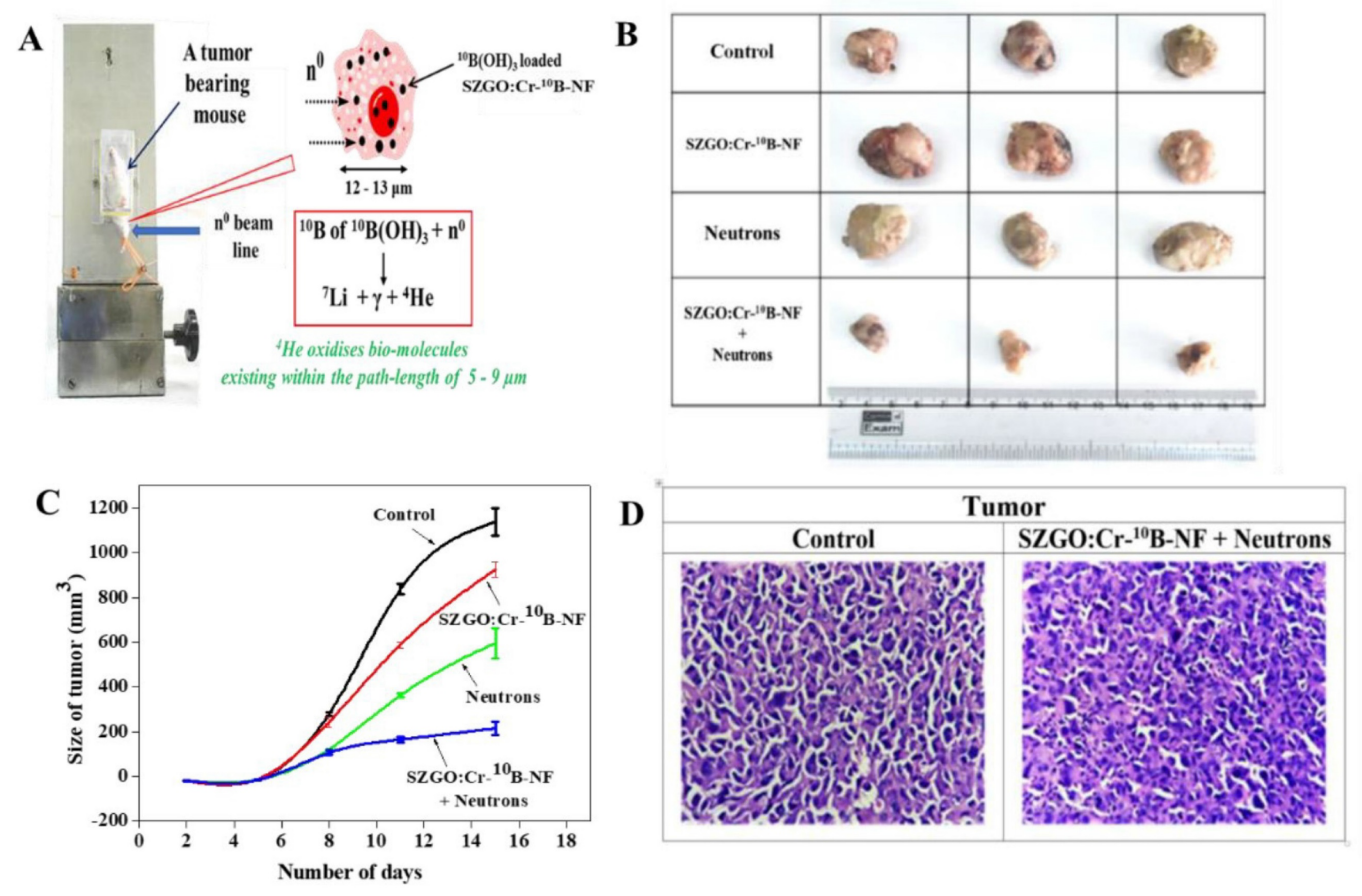
(A) Treatment of mice with SZGO: Cr-10B-NF; (B) photographic images of the tumor, after irradiation with neutron; (C) plot of size of tumor versus number of days; (D) histopathological studies of treated and control tumor. Reprinted (adapted) with permission from 99. Copyright {2024} American Chemical Society.

An innovative formulation was developed by amalgamating BNCT with silica coated Cr doped zinc gallate-based (SZGO: Cr-^10^B) persistent luminescent nanoparticles (PLNPs). PLNPs were prepared and conjugated with ^10^B(OH)3 and was again coupled with pH-(low)-insertion peptide (pHLIP). This pHLIP peptide aided in targeting the nanoformulation (SZGO: Cr-^10^B-NF) to the tumor cells. This peptide conjugated nanoformulation was tested against WEHI-164 cancer cells where the results showed substantial internalization of nanoformulation in the cancer cells and showed cytotoxicity with IC50 approximately 25 µΜ. Fibrosarcoma (WEHI-164) models were produced in BALB/c mice were used in the investigation of SZGO: Cr-^10^B-NF for the treatment of deeply seated tumors. The procedure was repeated twice which involved implanting fibrosarcoma tumor cells taken from a tumor-bearing mouse, using three mice in each trial. Mice were given a 200 μL solution containing 10 mg/mL of SZGO: Cr-^10^B-NF when the tumor size was around 0.5 cm, and they were exposed to neutron irradiation for 30 minutes (**Figure 4A**). Since SZGO: Cr-^10^B-NF takes 30 minutes to reach the tumor, the irradiation examines occurred 30 minutes to 1 hour after the intravenous injection. Photographic images demonstrated a 90% reduction in tumor volume when compared to control tumors (**Figure 4B**), and the tumor volume plotted over time provided confirmation of this (**Figure 4C**). The tumor volume in mice was significantly reduced by SZGO: Cr-^10^B-NF therapy and neutron irradiation, according to histopathological examinations. In comparison to untreated controls, treated mice showed decreased vascularity, fewer mitoses, and greater tumor necrosis (**Figure 4D**) 99.

Meher, N *et al.,* developed nanoparticles that target prostate-specific membrane antigen (PSMA), a protein expressed in high levels in prostate cancer cells. The nanoparticles are loaded with carborane and tested the ability of the nanoparticles to bind to PSMA and deliver their cargo to prostate cancer cells *in vitro*. The results showed that the nanoparticles were able to specifically target PSMA and deliver carborane to prostate cancer cells, leading to a significant reduction in cell viability. Furthermore, the study evaluated the nanoparticles *in vivo* using a mouse model of prostate cancer, where the nanoparticles were injected into mice with two groups of PSMA (-) PC3-Flu and PSMA (+) PC3-Pip. A 2-fold greater accumulation of nanoparticles was observed in PC3-Pip tumors as compared to PC3-Flu tumors. However, the study concluded that further research was needed to envision the benefits of this strategy 100.

A lipopeptide conjugated BSH was developed with an aim to develop lipopeptide-boron conjugates with high tumor selectivity, low toxicity, and increased cellular uptake for effective BNCT. The study described the synthesis of two different lipopeptide-Boron conjugates, LP1-BSH, and LP2-BSH. LP1-BSH contains a lysine-based lipopeptide, and LP2-BSH contains a cysteine-based lipopeptide. The researchers evaluated the physicochemical properties of the lipopeptide-boron conjugates, including their solubility, stability, and cytotoxicity. *In vitro* studies showed that both LP1-BSH and LP2-BSH conjugates had high cellular uptake, preferentially accumulating in tumor cells compared to normal cells. The lipopeptide-Boron conjugates demonstrated low toxicity and were well-tolerated by cells. LP2-BSH conjugates showed higher tumor selectivity and a more significant reduction in tumor volume than LP1-BSH conjugates. The conjugates demonstrated high tumor selectivity, and increased cellular uptake, making them effective for targeted BNCT 92.

## Conclusion

Peptide conjugated BNCT has the potential to become an important tool in the treatment of cancer. As research in this area continues, there are several future prospects for peptide conjugated BNCT. The critical issues related to boron delivery are inefficient delivery to the site of tumor tissues, which could be due to the lack of specificity and selectivity, leading to a low tumor-to-normal ratio. Additionally, there is variable microdistribution of boron, especially with evidence of variation in boron distribution among different subpopulations of tumor cells. Furthermore, there is a lag in evidence from clinical trials demonstrating the effectiveness of BNCT in treating tumor patients, necessitating further trials for clarification. These issues can be overcome by adopting active targeted delivery to the tumor tissue by linking boron to targeting ligands and implementing combination therapies, which include phototherapy and chemotherapy. Peptide conjugated BNCT has attracted much attention in enhancing the effective delivery to the tumor tissue. As more is learned about the proteins that are overexpressed on the surface of cancer cells, it may be possible to develop new targeting peptides that can be used in BNCT. These peptides may be more specific or more effective than current targeting peptides, leading to better treatment outcomes. Peptide conjugated BNCT may be used in combination with other cancer therapies, such as chemotherapy or immunotherapy 106,107. By combining therapies, it may be possible to improve treatment outcomes and overcome resistance to individual therapies. The boron-containing compounds used in peptide conjugated BNCT can also be used as imaging agents, allowing for the visualization of tumors before and during treatment. This may help to improve treatment planning and monitoring. It may also have applications in immunomodulation 108. By targeting specific cells or proteins, peptide conjugated BNCT may be able to selectively destroy these cells or modulate their activity. As technology continues to advance, it may be possible to improve the delivery of radiation in BNCT. For example, new neutron sources or boron-containing compounds may be developed that improve treatment outcomes or reduce side effects. Overall, the future prospects of peptide conjugated BNCT are promising, and continued research in this area may lead to new treatments and improved outcomes for patients with cancer and other diseases.

## Figures and Tables

**Table 1:** Clinical trials of BNCT compounds for various cancers

NCT Number	Status	Conditions	Interventions	Phases	Start date
NCT02759536	Unknown status	Melanoma|Boron Neutron Capture Therapy	Radiation: Boronophenylalanine and IHNI-based BNCT	Phase 1|Phase 2	2013
NCT02004795	Unknown status	Head and Neck Cancer|Recurrence	Radiation: BNCT + IG-IMRT	Phase 1|Phase 2	2013
NCT05737212	Recruiting	Recurrent High-grade Glioma|Recurrent Glioblastoma|Recurrent Anaplastic Astrocytoma|Recurrent Anaplastic Oligodendroglioma	Radiation: 500mg/kg/3hr followed by neutron irradiation to reach maximum brain dose of 9Gy-Eq|Radiation: 500mg/kg/3hr followed by neutron irradiation to reach maximum brain dose of 11Gy-Eq|Radiation: 500mg/kg/3hr followed by neutron irradiation to reach maximum brain dose of 13Gy-Eq	Phase 1|Phase 2	2022
NCT01173172	Completed	Head and Neck Cancer	Radiation: Boron Neutron Capture Therapy	Phase 1|Phase 2	2010
NCT00115440	Completed	Glioblastoma|Anaplastic Astrocytoma	Radiation: Bononophenylalanine (BPA)-based BNCT	Phase 1|Phase 2	2001
NCT00114790	Completed	Head and Neck Cancer	Radiation: boronophenylalanine-based BNCT	Phase 1|Phase 2	2003
NCT00927147	Terminated	Head and Neck Cancer	Radiation: BNCT|Drug: cetuximab|	Phase 1|Phase 2	2009
NCT04293289	Completed	Malignant Melanoma|Angiosarcoma	Other: CICS-1 (investigational device), SPM-011 (investigational drug)	Phase 1	2019
NCT00115453	Terminated	Glioblastoma	Radiation: irradiation	Phase 1|Phase 2	1999
NCT00002781	Unknown status	Melanoma (Skin)	Drug: boronophenylalanine-fructose complex	Phase 1	1996
NCT00085059	Terminated	Melanoma (Skin)	Drug: boronophenylalanine-fructose complex	Phase 2	2004
NCT00004015	Completed	Brain and Central Nervous System Tumors	Drug: sodium borocaptate|Procedure: adjuvant therapy	Phase 1	2002
NCT00974987	Completed	Brain and Central Nervous System Tumors	Radiation: BNCT (boron neutron capture therapy)|Radiation: XRT (X-ray radiation treatment) |Drug: TMZ (temozolomide)	Phase 2	2009
NCT05601232	Recruiting	Unresectable Angiosarcoma	Radiation: BNCT	Phase 2	2022
NCT01233492	Terminated	Brain and Central Nervous System Tumors	Drug: boron phenylalanine|Drug: mannitol|Other: biologic sample preservation procedure|Radiation: radiation therapy treatment planning/simulation	Phase 1	2007
NCT00059800	Completed	Melanoma (Skin)	Radiation: boron neutron capture therapy	Phase 2	2002
NCT00039572	Completed	Brain and Central Nervous System Tumors|Melanoma (Skin)|Metastatic Cancer	Drug: boronophenylalanine-fructose complex	Phase 1|Phase 2	2002

**Table 2:** Applications of peptide conjugated BNCT.

Peptide	BNCT compound	Cancer	Ref
Z33 peptide, which shows specific recognition of and interaction with the Fc domain of human IgG, for on-demand receptor targeting	Disodium mercaptoundecahydrododecaborate (BSH)	Human epidermoid carcinoma derived A431 cells	84
IFLLWQR (IF7)	BSH	MBT2 bladder tumor-bearing mice	85
[D-Phe6, β-Ala11, Ala13, Nle14]Bn(6-14) (sBB2L)	Bis-deoxygalactosyl-carborane	HEK293 cells	83
[F7 ,P34]-NPY	Bis-deoxygalactosyl-carborane	MCF-7, humanderived breast cancer cell line	86
Octa-arginine (R8)	Disodium mercaptoundecahydrocloso-dodecaborate	C6 glioma cells	87
Translocator protein (TSPO) ligand	Dodecaborate-based pyrazolopyrimidine	Breast cancer	88
A6K peptide	Mercaptoundecahydrododecaborate (BSH)	U87 delta EGFR glioma cell	9
Integrin-binding cyclic RGD peptide (GPU-201)	Mercaptododecaborate-10B (BSH)	U87MG and SCC VII cells	89
iRGD	BSH	A549 cells	90
Cyclic RGD (cRGD) peptide	Closo-Dodecaborate Albumin	U87MG and A549 cells	91
Lipopeptide	BSH	T98G glioblastoma cells	92
RGD	Closo-dodecaborate-(Ga-DOTA)	U-87 MG human glioblastoma cells	93
Cyclic arginine-glycine-aspartate (cRGD)	Closo-dodecaborate albumin conjugate (MID-AC) with albumin	F98, C6 glioma, and 9L gliosarcoma cells	94
Tyrosine	BPA-containing dipeptides	AsPC-1 cells (pncreatic cancer model in mice)	95
Poly-arginine peptide	BSH	Human glioma cell lines U87MG and U251MG, human breast cancer cell lines MCF7 and MDA-MB-231, human pancreatic cancer cell lines CFPAC1 and PANC1, and murine mammary gland cell line NMuMG	96
Hexapeptide ghrelin receptor agonist	Meta-carborane	HEK293_GhrR_Eyfp cells	97
NapKYpF	BPA	HeLa cells	98
pH-(low)-insertion peptide (pHLIP)	10B(OH)3	WEHI-164 cancer cells, melanoma-induced C57BL/6 and fibrosarcoma-induced BALB/c mice	99
Prostate-specific membrane antigen	Carborane	PC3-Pip cells	100
